# Supplementary material for: Does Sodium Intake Induce Systemic Inflammatory Response? A Systematic Review and Meta-Analysis of Randomized Studies in Humans
Source: Nutrients. 2021 Jul 30;13(8):2632. doi: 10.3390/nu13082632 (PMC8399701; doi:10.3390/nu13082632)

Supplementary material

Does sodium intake induce systemic inflammatory response?

A systemic review and meta-analysis of randomized studies in humans

Eirini D. Basdeki[1,2], Anastasios Kollias[3], Panagiota Mitrou [4], Christiana Tsirimiagkou[1,2], Marios K. Georgakis[5], Antonios Chatzigeorgiou[6], Antonios Argyris[1], Kalliopi Karatzi[7], Yannis Manios[2], Petros P. Sfikakis[8], Athanase D. Protogerou[1]

[1]Cardiovascular Prevention & Research Unit, Clinic & Laboratory of Pathophysiology, Department of Medicine, National and Kapodistrian University of Athens, Greece

[2]Department of Nutrition and Dietetics, School of Health Science and Education, Harokopio University of Athens, Greece

[3]Hypertension Center STRIDE-7, National and Kapodistrian University of Athens, School of Medicine, Third Department of Medicine, Sotiria Hospital, Athens, Greece

[4]Hellenic Republic Ministry of Health, Athens, Greece

[5]Institute for Stroke and Dementia Research, University Hospital, LMU Munich, Germany

[6]Department of Physiology, Medical School, National & Kapodistrian University of Athens, Athens, Greece.

[7]Department of Food Science and Human Nutrition, Agricultural University of Athens, Greece

[8]1st department of Propaedeutic and Internal Medicine, Athens University Medical School, Laiko Hospital, Athens, Greece

Corresponding author:

Professor Athanase D Protogerou

Cardiovascular Prevention & Research Unit,

Clinic & Laboratory of Pathophysiology,

Department of Medicine,

National and Kapodistrian University of Athens,

75, Mikras Asias Street, (Building 16, 3rd floor, room 8),

115 27 Athens, GR,

Tel/fax: 0030 210 746 2566, email: aprotog@med.uoa.gr

Table S1. Prisma Checklist

Section/topic	#	Checklist item	Reported on page #
**TITLE**			
Title	1	Identify the report as a systematic review, meta-analysis, or both.	1
**ABSTRACT**			
Structured summary	2	Provide a structured summary including, as applicable: background; objectives; data sources; study eligibility criteria, participants, and interventions; study appraisal and synthesis methods; results; limitations; conclusions and implications of key findings; systematic review registration number.	2
**INTRODUCTION**			
Rationale	3	Describe the rationale for the review in the context of what is already known.	3
Objectives	4	Provide an explicit statement of questions being addressed with reference to participants, interventions, comparisons, outcomes, and study design (PICOS).	3
**METHODS**			
Protocol and registration	5	Indicate if a review protocol exists, if and where it can be accessed (e.g., Web address), and, if available, provide registration information including registration number.	4
Eligibility criteria	6	Specify study characteristics (e.g., PICOS, length of follow-up) and report characteristics (e.g., years considered, language, publication status) used as criteria for eligibility, giving rationale.	4
Information sources	7	Describe all information sources (e.g., databases with dates of coverage, contact with study authors to identify additional studies) in the search and date last searched.	4
Search	8	Present full electronic search strategy for at least one database, including any limits used, such that it could be repeated.	4
Study selection	9	State the process for selecting studies (i.e., screening, eligibility, included in systematic review, and, if applicable, included in the meta-analysis).	5
Data collection process	10	Describe method of data extraction from reports (e.g., piloted forms, independently, in duplicate) and any processes for obtaining and confirming data from investigators.	5
Data items	11	List and define all variables for which data were sought (e.g., PICOS, funding sources) and any assumptions and simplifications made.	4
Risk of bias in individual studies	12	Describe methods used for assessing risk of bias of individual studies (including specification of whether this was done at the study or outcome level), and how this information is to be used in any data synthesis.	5
Summary measures	13	State the principal summary measures (e.g., risk ratio, difference in means).	5

Synthesis of results	14	Describe the methods of handling data and combining results of studies, if done, including measures of consistency (e.g., I^2) for each meta-analysis.	5
**Section/topic**	**#**	**Checklist item**	**Reported on page #**
Risk of bias across studies	15	Specify any assessment of risk of bias that may affect the cumulative evidence (e.g., publication bias, selective reporting within studies).	5
Additional analyses	16	Describe methods of additional analyses (e.g., sensitivity or subgroup analyses, meta-regression), if done, indicating which were pre-specified.	5
**RESULTS**			
Study selection	17	Give numbers of studies screened, assessed for eligibility, and included in the review, with reasons for exclusions at each stage, ideally with a flow diagram.	6
Study characteristics	18	For each study, present characteristics for which data were extracted (e.g., study size, PICOS, follow-up period) and provide the citations.	6
Risk of bias within studies	19	Present data on risk of bias of each study and, if available, any outcome level assessment (see item 12).	9
Results of individual studies	20	For all outcomes considered (benefits or harms), present, for each study: (a) simple summary data for each intervention group (b) effect estimates and confidence intervals, ideally with a forest plot.	7
Synthesis of results	21	Present results of each meta-analysis done, including confidence intervals and measures of consistency.	9, 10
Risk of bias across studies	22	Present results of any assessment of risk of bias across studies (see Item 15).	9
Additional analysis	23	Give results of additional analyses, if done (e.g., sensitivity or subgroup analyses, meta-regression [see Item 16]).	9, 10
**DISCUSSION**			
Summary of evidence	24	Summarize the main findings including the strength of evidence for each main outcome; consider their relevance to key groups (e.g., healthcare providers, users, and policy makers).	11, 12
Limitations	25	Discuss limitations at study and outcome level (e.g., risk of bias), and at review-level (e.g., incomplete retrieval of identified research, reporting bias).	13
Conclusions	26	Provide a general interpretation of the results in the context of other evidence, and implications for future research.	13
**FUNDING**			
Funding	27	Describe sources of funding for the systematic review and other support (e.g., supply of data); role of funders for the systematic review.	Not available

Table S2. Assessment of risk of bias

	Random sequence generation	Allocation concealment	Blinding of participants and researchers	Blinding of outcome assessment	Incomplete outcome data	Selective reporting	Other bias (sodium assessment method)
Parrinello G. (2009) [38]	+	?	+	+	+	+	?
Forrester G. (2010) [36]	+	?	+	+	+	+	+
Mallamaci F. (2013) [27]	+	?	-	+	+	+	+
Campbell K. (2014) [40]	+	?	+	+	+	+	+
Telini L. (2014) [35]	+	?	?	+	+	+	?
Wenstedt E. (2019) [39]	+	?	-	+	+	+	+

**Key**	
Low risk of bias	+
High risk of bias	-
Unclear risk of bias	?

Figures S1a, b, c. Begg's Funnel Plot for CRP, TNF-a & IL-6

S1a

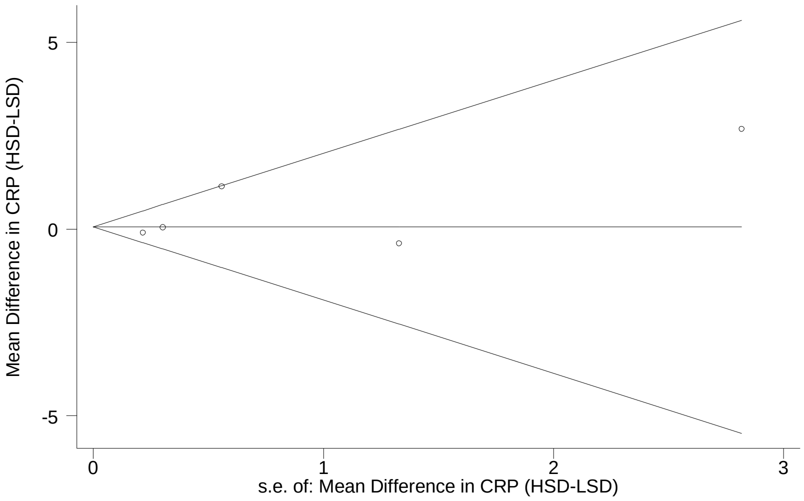

S1b

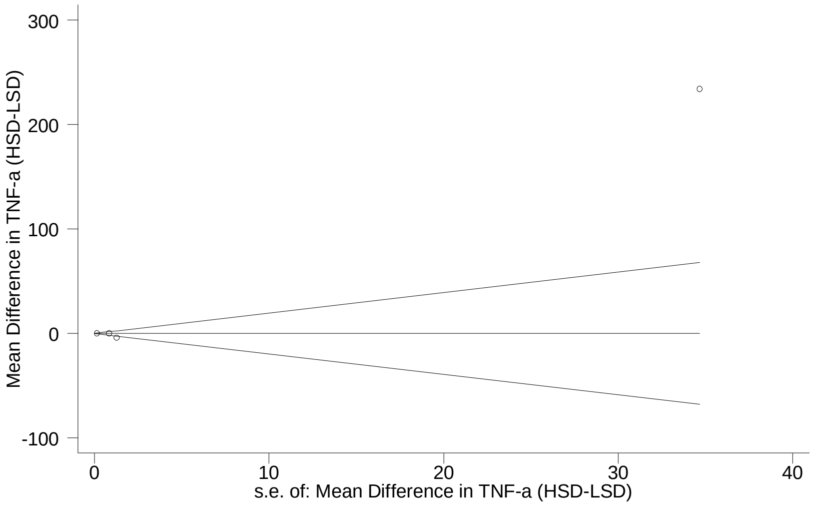

S1c

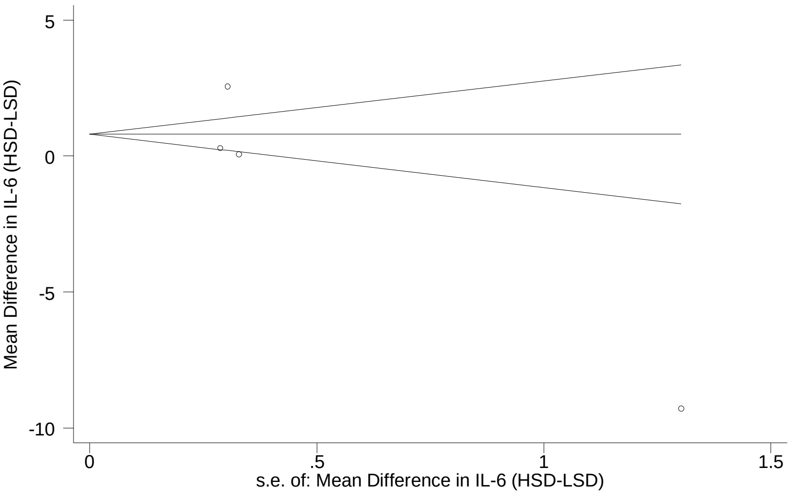
5
0
-5
-10
Mean Difference in IL-6 (HSD-LSD)
0
.5
1
1.5
s.e. of: Mean Difference in IL-6 (HSD-LSD)

Figure S2. Begg's Funnel Plot for SPB & DBP.

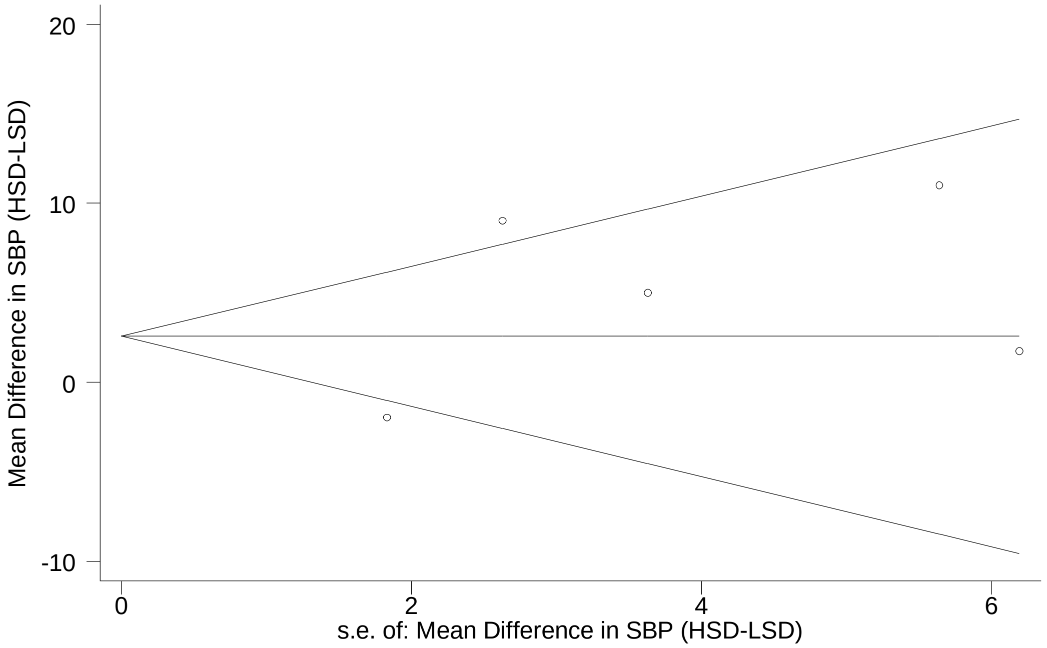

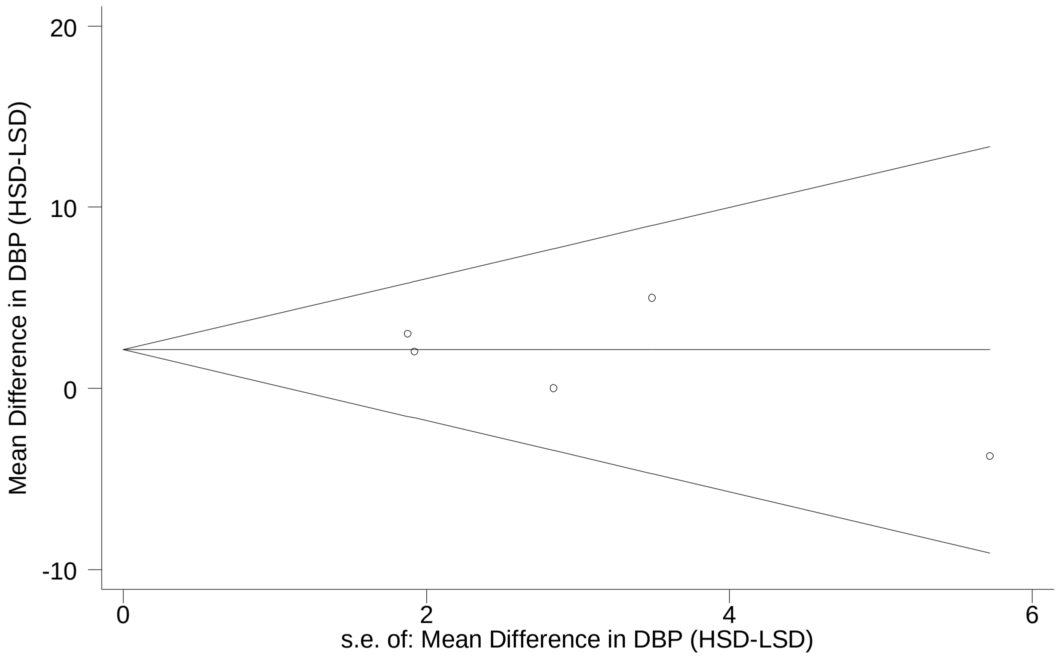

Figure S3. Metaregression analysis for SBB & DBP and duration of the intervention.

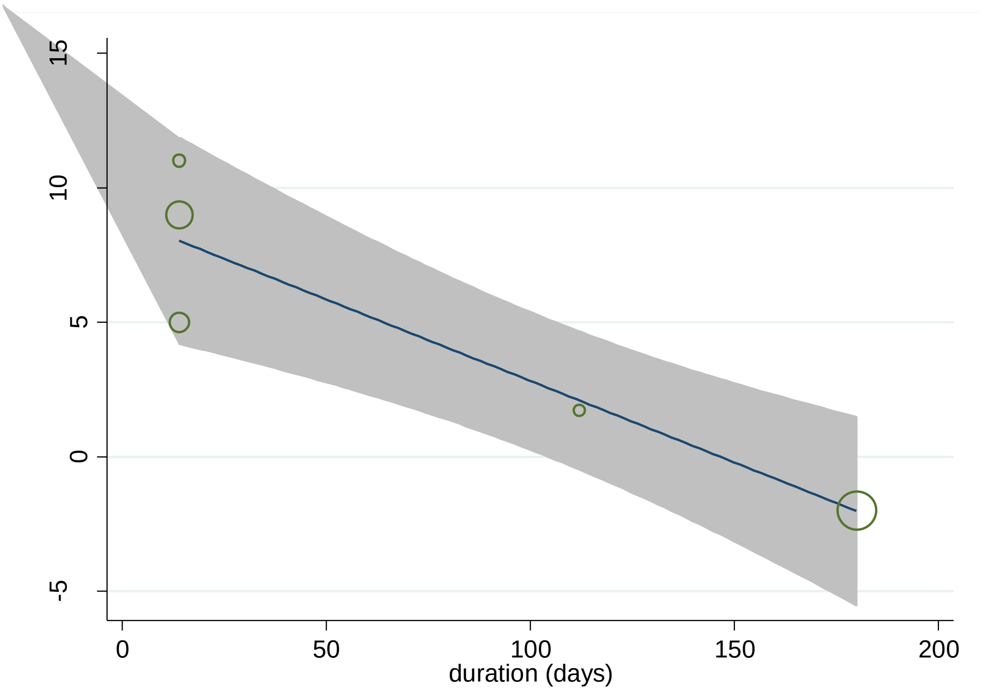

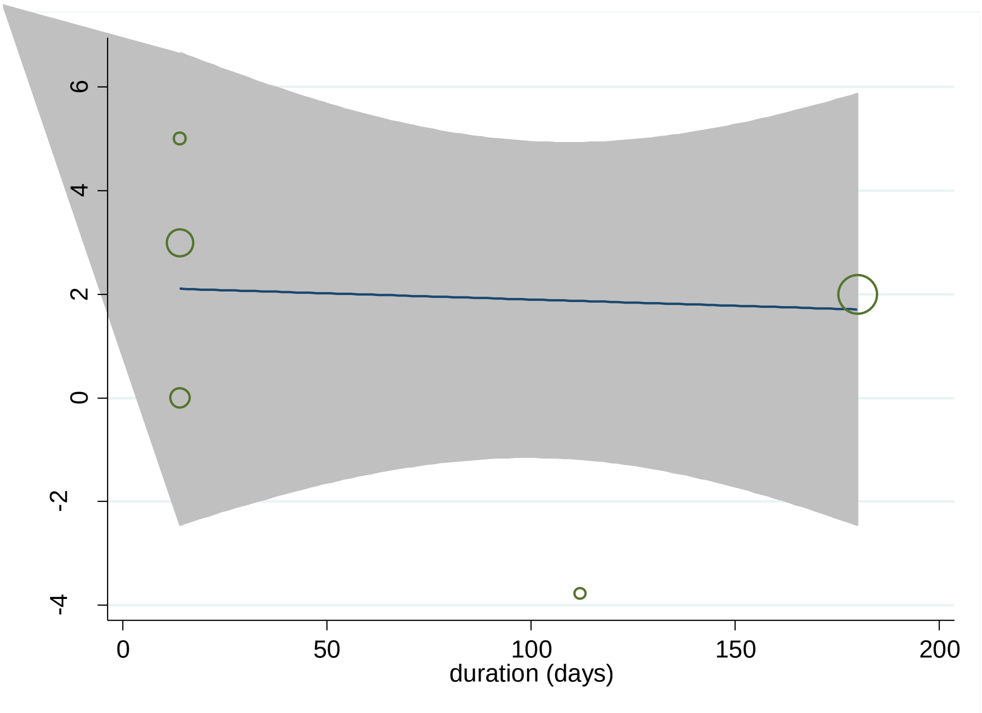

Supplement: Supplementary file 1 [file nutrients-13-02632-s001.zip › nutrients-1259706-SI.pdf]
